# Vulvovaginal candidiasis and vaginal microflora interaction: Microflora changes and probiotic therapy

**DOI:** 10.3389/fcimb.2023.1123026

**Published:** 2023-02-03

**Authors:** Zhongwen Sun, Xinnuo Ge, Bo Qiu, Ze Xiang, Chun Jiang, Jian Wu, Yuan Li

**Affiliations:** ^1^ Department of Medical Technology, Suzhou Vocational Health College, Suzhou, Jiangsu, China; ^2^ Zhejiang University School of Medicine, Hangzhou, Zhejiang, China; ^3^ State Key Laboratory for Diagnosis and Treatment of Infectious Diseases, National Clinical Research Center for Infectious Diseases, Collaborative Innovation Center for Diagnosis and Treatment of Infectious Diseases, The First Affiliated Hospital, Zhejiang University School of Medicine, Hangzhou, Zhejiang, China; ^4^ Department of Clinical Laboratory, The Affiliated Suzhou Hospital of Nanjing Medical University, Suzhou Municipal Hospital, Gusu School, Nanjing Medical University, Suzhou, Jiangsu, China; ^5^ Departments of Cardiology, The Affiliated Suzhou Hospital of Nanjing Medical University, Suzhou Municipal Hospital, Gusu School, Nanjing Medical University, Suzhou, Jiangsu, China

**Keywords:** vulvovaginal candidiasis, vaginal infection, microbial changes, *Lactobacillus*, treatment

## Abstract

Vaginal microbiome is mutually beneficial to the host and has a significant impact on health and disease. *Candida* species, including *Candida albicans*, are part of the mucosal flora of most healthy women. Under suitable conditions, they can live in the vulvovaginal mucosa, resulting in symptomatic vulvovaginal candidiasis (VVC). Based on the analysis of 16S ribosomal RNA gene sequences, great progress has been made in exploring the composition and structure of vaginal bacterial community. Moreover, researchers have conducted several studies on whether vaginal microbiome will change during VVC infection. In addition, it has been reported that vaginal colonization of probiotics in vaginal microorganisms, especially *Lactobacillus*, can effectively reduce the risk of VVC and treat VVC. This review aims to summarize the changes of vaginal microflora during VVC infection, and further point out the possibility of using lactic acid bacteria as probiotics to treat VVC, so as to reduce the adverse consequences of VVC infection and reduce the expensive treatment cost.

## Introduction

Bacteria are the largest group of all organisms, which first appeared about 3.8 billion years ago, and nucleolus is one of their most obvious signs (Mojzsis et al., 1996). Fungi have true nuclei and complete organelles, so they are also called eukaryotic cell type microorganisms (Lopez-Garcia and Moreira, 2020). The human microbiome is made up of archaea, bacteria, viruses, and fungi that form a highly complex web of interactions with each other and their hosts (Wang et al., 2015; Wu et al., 2020). The host and the microbial community have co-evolved an immune system to prevent the colonization of the foreign microorganisms in the body (Wu et al., 2019). Researchers have used a variety of methods, including metabolomics, proteomics, transcriptomics and metagenomics, to verify that microbial communities are dynamic, interactive and complex organic whole (Chen et al., 2021; Xiang et al., 2022).

The impact of microbial flora in the human body has aroused extensive concern. It not only plays an important role in the intestine, which is widely studied nowadays, but also gradually functions in other organ systems (Champer et al., 2018; Khanna et al., 2022). Nowadays, researchers pay more and more attention to women’s health, especially the vaginal microbiota, since the vagina has a huge microecosystem containing billions of species of microorganisms (Witkin and Forney, 2020). In recent years, tremendous advances have been made in exploring the composition and structure of vaginal bacterial community using the method based on analysis of 16S ribosomal RNA (rRNA) gene sequences (Chen et al., 2017). In the case of bacterial or fungal infections, changes in the vaginal microbiome have also been reported (Onderdonk et al., 2016; Chen et al., 2021).

In addition to the widely studied bacterial vaginosis (BV), vulvovaginal candidiasis (VVC) has been a hot topic, which is a multifactor infectious disease of women’s lower reproductive tract, mainly caused by *Candida albicans*, resulting in pathological inflammation (Farr et al., 2021). In a study that collected 649 separate strains of VVC patients in China, researchers found that *Candida albicans* was the dominant pathogen of VVC, but the proportion of non-*Candida albicans* infection was also increasing (Pang et al., 2022).

This review aims to describe changes in the vaginal microbiome during VVC, propose the possible associations between VVC and vaginal microbiome changes, and summarize the possibility of using this association to treat and defend VVC to reduce adverse health outcomes and treatment costs.

## Vaginal microbiota in healthy women

The native microbiota in the vaginal environment is thought to be symbiotic with the host (Ma et al., 2012). The technology for assessing human microbial diversity has gradually progressed. Nowadays, scientists have successfully identified different bacterial communities in the vagina of women of four races using advanced high-throughput methods and analyzed species composition through 16S rRNA gene sequencing (Di Bella et al., 2013). The vaginal bacterial communities of these women can be roughly divided into five types: the first four are mainly composed of *Lactobacillus crispatus*, *Lactobacillus gasseri, Lactobacillus iners, and Lactobacillus jensenii*, while the fifth bacterial community is relatively specific, showing a low proportion of *Lactobacillus* and a high proportion of strictly anaerobic organisms (Ravel et al., 2011). Therefore, *Lactobacillus* are considered the most common microorganisms isolated from the vagina of healthy people, including *Lactobacillus iners*, *Lactobacillus crispatus*, *Lactobacillus gasseri* and *Lactobacillus jensenii* (Chee et al., 2020). In several studies, these vaginal *Lactobacillus* can be used to prevent the invasion of pathogens (Petrova et al., 2017; Kalia et al., 2020).

In addition, fungi, especially *Candida*, are believed to exist in the vaginal mucosa as symbiotes, forming a complex vaginal ecosystem with other bacteria (Gow and Hube, 2012; Hall and Noverr, 2017). The defense function of fungi plays a role through various mechanisms, including lowering vaginal pH, producing bioactive compounds, competing for nutrients and adhesion sites, and regulating host immune responses (Parolin et al., 2015; Calonghi et al., 2017; Parolin et al., 2018; Younes et al., 2018).

Of course, it cannot be ignored that the vaginal microbiome is variable. Vaginal microbiome varies among individuals, and these differences are caused by differences in sexual habits, menstrual hygiene habits (Noyes et al., 2018), flushing habits (Schwebke et al., 1999), chronic stress (Culhane et al., 2002), geography (Gupta et al., 2017), socioeconomic status, psychosocial pressure, community characteristics (Paul et al., 2008), and other factors. Moreover, vaginal pH also varies due to the different compositions in certain populations. Recent studies have found that a small percentage of asymptomatic healthy women have low levels of *Lactobacillus* in their vaginas, but they include a variety of facultative or strictly anaerobic bacteria with a slightly higher pH (5.3-5.5), which is similar to the fifth bacterial community described above. When these women did not have the disease, their vaginal bacterial community was considered normal, while the abnormal type of vaginal microbiome may be strongly associated with symptomatic bacterial vaginosis (Zhou et al., 2007; Zhou et al., 2010; Denning et al., 2018). Furthermore, vaginal microorganisms are a dynamic ecosystem of more than 200 bacteria, and the same individuals may also be significantly different from their previous performance (Chen et al., 2017). There is some evidence that the vaginal microbiome changes throughout a woman’s life and therefore has an important impact on the quality of life from newborn to post-menopausal age.

The changes of the vaginal microbiome include the decrease in the abundance of *Lactobacillus* and the increase in the abundance of facultative and anaerobic organisms, which can make the host vulnerable to a variety of diseases, such as low birth weight and increased bacterial infection risk (Jayaram et al., 2020). Therefore, the physiological state of the vaginal environment is of great significance for the health and reproduction of the host (Amabebe and Anumba, 2018).

To sum up, based on the variable and important characteristics of vaginal microbiome, we strongly advocate a more precise definition of the bacterial community in healthy women, which should fully consider the differences among individuals. The precise definition of a healthy vaginal microbiome can be used to diagnose disease faster and more accurately when the vaginal microbiome changes.

## Vaginal microbial changes during VVC

The disruption of vaginal ecosystem balance can cause pathogen overgrowth, which will lead to more complex vaginal infections, such as BV, sexually transmitted infections (STIs), and VVC (Chee et al., 2020). Among them, VVC is defined as a symptom of inflammation and excessive overgrowth of *Candida*, especially *Candida albicans* (Sobel, 2007). *Candida albicans* is a major species in premenopausal, pregnant, and acute VVC women (Farr et al., 2021). VVC is one of the most common infectious vaginitis, second only to BV, and it is estimated that about 75% of women have been infected at least once in their lifetime. In addition, recurrent VVC can affect nearly 8% of women in the world (Sobel, 2016; Matheson and Mazza, 2017; Denning et al., 2018). Many experiments have shown that vaginal microbiota changes, including species and metabolites, occur during VVC infection.

Some experiments showed no significant change in the vaginal microbiota during VVC infection. As early as 2009, studies showed that no new bacteria were found in women who frequently suffered from VVC. This study evaluated the vaginal microbial species composition of 42 women with and without frequent VVC, and evaluated microbial community diversity on this basis. The results showed no significant differences in the vaginal microbiome between the two groups. This study also showed that the vaginal flora of most women in the two groups was dominated by *Lactobacillus*, which was similar to the vaginal microbiota of most healthy women mentioned above. Therefore, it failed to provide evidence to prove the change or unusual presence of the vaginal bacterial communities in women with frequent VVC compared to those without frequent VVC (Zhou et al., 2009).

**Figure 1:**
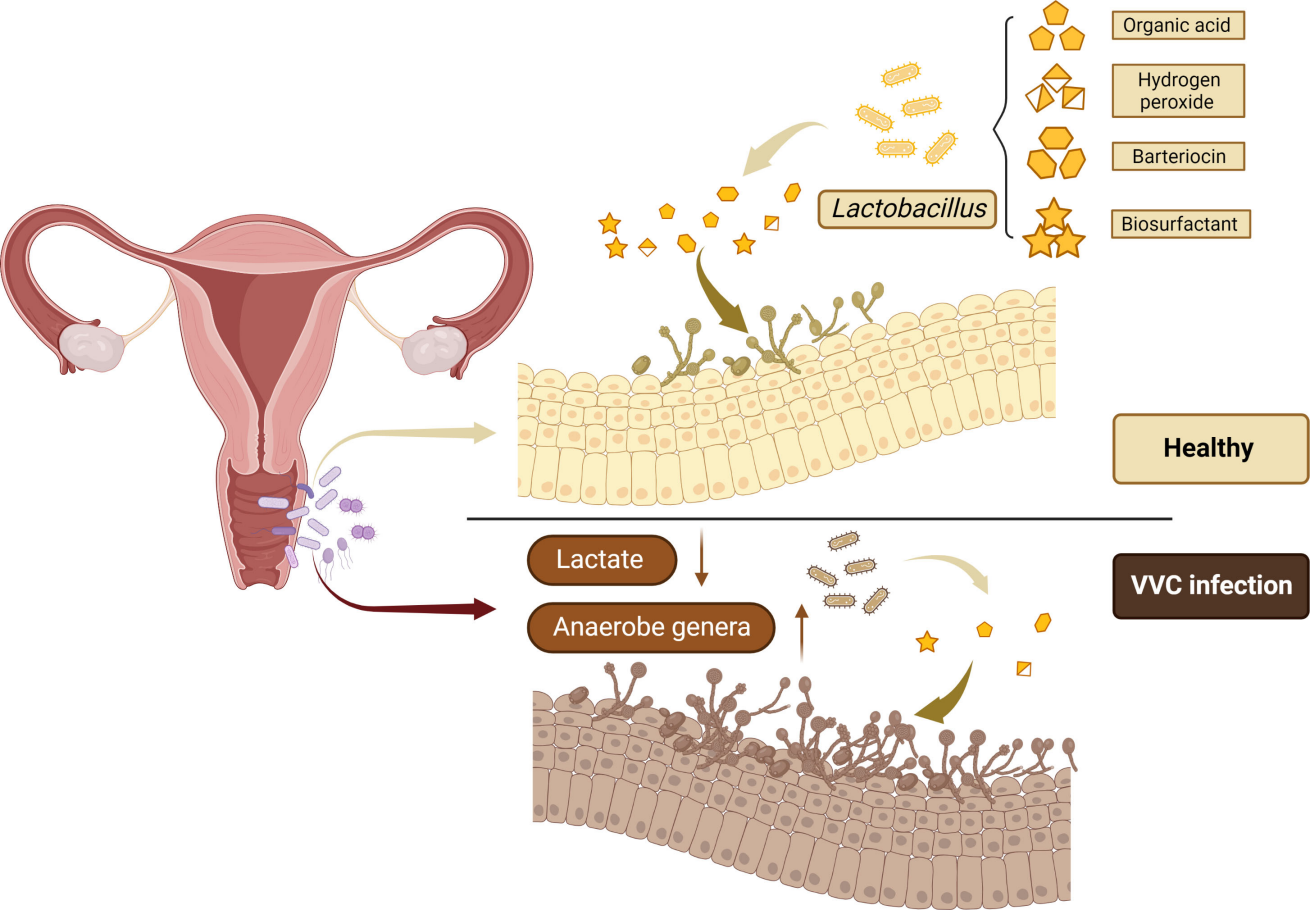
*Lactobacillus* is the most frequently isolated microorganism from the healthy women vagina to prevent the colonization and overgrowth of pathogens by excreting metabolic by-products, including organic acid, hydrogen peroxide, bacteriocin, and biosurfactant.

However, there are still many studies to support the theory that the vaginal microbiome of VVC patients is different from that of the normal population (**Figure 1**). In 1980, researchers studied *Candida albicans* in the vaginal microbiome. The results obtained from 340 vaginal specimens showed in the absence of *Candida albicans*, all microbiome increased, especially Gram-negative bacteria (Auger and Joly, 1980). Liu et al. measured the vaginal microbial communities in patients with BV and VVC. The results showed that VVC patients had high changes in their vaginal microbiome (Liu et al., 2013). The healthy vaginal microbiome is dominated by *Lactobacillus crispatus*, however, when turning from health to Chlamydia trachomatis infection (CT), VVC, and BV, *Lactobacillus crispatus* is gradually replaced by *Lactobacillus iners*. Studies have shown that CT, VVC and BV are mainly characterized by *Gardnerella, Prevotella, Megasphaera, Roseburia*, and *Atopobium*. At the same time, changes in bacterial community during genital infection will lead to significant changes in the composition of vaginal metabolites. The production of lactic acid is highly conserved in the vaginal microorganisms among different women, while the decline of lactic acid is a common sign of all the above pathological conditions (Ceccarani et al., 2019).

The microbiome of many VVC patients receiving treatment is similar to the abnormal vaginal microbiome of healthy women, suggesting that the abnormal vaginal microbiome may constitute a transitional state between disease and health, especially since many women have asymptomatic infections with *Candida* (Liu et al., 2013). Therefore, in addition to focusing on women with abnormal vaginal microbiome during VVC, we should also pay attention to women with asymptomatic infections with *Candida*, and women who appear healthy but have abnormal vaginal microbiome.

## *Lactobacillus* treatment and defense against VVC

Over the past few years, the investigation of the vaginal microbiome has grown exponentially. These studies together found that a dominant microflora was observed in healthy vaginas: *Lactobacillus*. Therefore, it is proposed that vaginal colonization of *Lactobacillus* can reduce the risk of VVC.

It has been proved that the bacterial microbiome in the mucosal layer can achieve defense function through acidic pH regulation, release antifungal peptides and physiological control of ecological disorders. The important role of bacterial microorganisms, especially *Lactobacillus*, in maintaining vaginal health can promote their application as potential therapeutic methods for VVC, and alleviate the symptoms of VVC (Balakrishnan et al., 2022). *Lactobacillus* are thought to prevent the colonization and overgrowth of pathogens by excreting metabolic by-products and acidification of the vaginal microenvironment, helping maintain body balance. Metabolites of *Lactobacillus*, including organic acid, hydrogen peroxide, bacteriocin and biosurfactant, can contribute to antifungal effect (Wang et al., 2017; Fuochi et al., 2019). Researchers are also exploring whether *Lactobacillus* can prevent pathogens from colonizing the body, and whether it can be used to treat VVC in women. In recent years, several studies on the influence of *Lactobacillus* on VVC have drawn different conclusions, mainly regarding the role of *Lactobacillus* in preventing *Candida* colonization and treating VVC.

*In vitro* experiments have shown that some *Lactobacillus* strains can inhibit the adhesion and growth of *Candida albicans*, which will provide new insights into the prevention and treatment of VVC (**Table 1**). As early as 1999, it was reported that *Lactobacillus pentosus TV35b*, isolated from the vaginal posterior fornix secretion of prenatal patients, produced a bacteriocin like peptide to inhibit *Candida albicans* (Okkers et al., 1999). In 2001, Osset et al. found that 8 of the 15 *Lactobacillus* significantly blocked the adhesion of *Candida albicans* Y18 to vaginal cells. In liquid assays, some *Lactobacillus* had a certain degree of inhibition to *Candida albicans* Y17 (Osset et al., 2001). In 2005, Strus et al. found that *Lactobacillus delhalis* produced a large amount of H_2_O_2_, which more strongly inhibited *Candida albicans* growth faster than other strains isolated from healthy women vaginas, and *Lactobacillus plantarum* without H_2_O_2_ showed the longest inhibitory activity after 24 h (Strus et al., 2005). In 2016, researchers evaluated the *in vitro* probiotic potential of 23 *Lactobacillus* isolated from the vaginal ecosystem of healthy women for BV and VVC treatment. *In vitro* experiments have shown that all these strains had excellent adhesion properties, which can aggregate with *Gardnerella vaginalis* and *Candida albicans*, producing a large amount of hydrogen peroxide and lactic acid. These results suggested that these strains have promising probiotic potential for the prevention and treatment of BV and VVC (Santos et al., 2016). In 2017, Wang et al. found that *Lactobacillus crispatus* showed significant antimicrobial activity. Seven kinds of cell-free supernatants from the *Lactobacillus crispatus* reduced the growth of *Candida albicans*. It was shown that *Lactobacillus crispatus* is a dominant *Lactobacillus* genus, which is associated with a healthy vagina and strongly inhibits the growth and hyphal formation of *Candida albicans* (Wang et al., 2017). In 2019, Li et al. investigated the therapeutic effects and mechanisms of *Lactobacillus crispatus* and *Lactobacillus delbrueckii* on VVC caused by *Candida albicans* in a Sprague-Dawley rat model. *In vitro* results demonstrated that two *Lactobacillus* strains showed inhibitory activity against *Candida* colony forming unit counts, indicating that *Lactobacillus crispatus* and *Lactobacillus delbrueckii* may become potential adjuvants of VVC, especially in patients with antifungal drug resistance, adverse reactions or contraindications (Li et al., 2019). Some *Lactobacillus* species can produce small molecules under laboratory conditions that can block *Candida albicans* yeast-to-filament transition, which is an important virulence trait. In 2021, relevant results showed that the 1-acetyl-β-carboline produced by *Lactobacillus* can prevent the yeast-to-filament transition of *Candida albicans* by inhibiting Yak1 (MacAlpine et al., 2021). Researchers examined the inhibitory activity of bacteriocin-like inhibitory substances (BLISs) from *Lactobacillus* and *Streptococci* on *Candida albicans* and non-*Candida albicans* isolated from patients with VVC. Using agar pore diffusion test, BLISs can inhibit both *Candida albicans* and non-*Candida albicans* (Hefzy et al., 2021). The bactericidal effect of *Lactobacillus casei* on the main VVC pathogenic species *Candida albicans, Candida tropicalis, Clostridium novigen and Paracandida* was investigated by calculating the colony forming units after co-cultivation. *Lactobacillus casei* had an inhibitory effect on all tested *Candida* genera, and *Lactobacillus casei* could reduce the formation of *Candida albicans* mycelia and early biofilm, showing a strong anti-*Candida* effect (Paniagua et al., 2021).

**Table 1:** *In vitro* experiments.

Authors	Time	Results	References
Okkers et al.	1999	*Lactobacillus pentosus TV35b* produced a bacteriocin like peptide to inhibit *Candida albicans*	(Okkers et al., 1999)
Osset et al.	2001	Some *Lactobacillus* had a certain degree of inhibition to *Candida albicans* Y17.	(Osset et al., 2001)
Strus et al.	2005	*Lactobacillus delhalis* produced a large amount of H2O2 and inhibited *Candida albicans* growth. *Lactobacillus plantarum* without H_2_O_2_ showed the longest inhibitory activity after 24 h	(Strus et al., 2005)
Santos et al.	2016	*Lactobacillus* strains had excellent adhesion properties, which can aggregate with *Gardnerella vaginalis* and *Candida albicans*, producing a large amount of hydrogen peroxide and lactic acid.	(Santos et al., 2016)
Wang et al.	2017	7 kinds of cell-free supernatants from the *Lactobacillus crispatus* reduced the growth of *Candida albicans*.	(Wang et al., 2017)
Li et al.	2019	*Lactobacillus* strains showed inhibitory activity against *Candida* colony forming unit counts, indicating that *Lactobacillus crispatus* and *Lactobacillus delbrueckii* may become potential adjuvants of VVC.	(Li et al., 2019)
MacAlpine et al.	2021	1-acetyl-β-carboline produced by *Lactobacillus* can prevent the yeast-to-filament transition of *Candida albicans* by inhibiting Yak1.	(MacAlpine et al., 2021)
Hefzy et al.	2021	Bacteriocin-like inhibitory substances can inhibit both *Candida albicans* and non-*Candida albicans.*	(Hefzy et al., 2021)
Paniagua et al.	2021	*Lactobacillus casei* had an inhibitory effect on all tested *Candida* genera, and *Lactobacillus casei* could reduce the formation of *Candida albicans* mycelia and early biofilm.	(Paniagua et al., 2021)

*Lactobacillus* strains and their products can inhibit the growth of *Candida*, and clinical studies are also in progress (**Table 2**). In 2001, a trial of 64 healthy women was conducted in which oral capsules of *Lactobacillus rhamnosus* GR-1 and *Lactobacillus fermentum* RC-14 were taken daily. The results showed that the combination of *Lactobacillus rhamnosus* GR-1 and *Lactobacillus fermentum* RC-14 can not only be safely used for daily use in healthy women, but also reduce the potential pathogenic bacteria and yeast colonization in vagina (Reid et al., 2003). De Seta et al. also evaluated the effect of the application of *Lactobacillus plantarum* P17630 on the recovery of vaginal microbiota and prevention of recurrence in women with acute VVC. These results confirmed the role of *Lactobacillus plantarum* P17630 as a potential empirical preventive agent, which can reduce vaginal discomfort after routine treatment of acute VVC and improve vaginal pH (De Seta et al., 2014). In 2019, Russo et al. found that the combination of a *Lactobacillus* mixture with lactoferrin is a safe and effective auxiliary method to reduce VVC symptoms and recurrence (Russo et al., 2019). Based on *in vitro* evaluation, Oerlemans et al. selected three strains from the *Lactobacillus* genus complex (*Lactobacillus rhamnosus GG, Lactobacillus pentosus KCA1*, and *Lactobacillus plantarum WCFS1*) and prepared them with gel for vaginal use. The gel was evaluated in 20 patients suffering from acute VVC, whose fungal concentrations were similar to those of women treated with fluconazole. These results pointed out the important aspects of choosing *Lactobacillus* for VVC treatment in the future (Oerlemans et al., 2020). Besides, the safety and antimicrobial activity of biosurfactants (BS) isolated from *Lactobacillus* vaginalis strains on *Candida* genus were studied. The results showed that BS from *Lactobacillus crispatus* BC1 can interfere with the adherence of *Candida in vivo* and *in vitro*, indicating its potential as a preventive measure against *Candida* mucosal damage during VVC (De Gregorio et al., 2020).

**Table 2:** Clinical studies.

Authors	Time	Results	References
Reid et al.	2001	The combination of *Lactobacillus rhamnosus* GR-1 and *Lactobacillus fermentum* RC-14 can not only be used for daily use in healthy women, but also reduce the potential pathogenic bacteria and yeast colonization in vagina.	(Reid et al., 2003)
De Seta et al.	2014	*Lactobacillus plantarum* P17630 can reduce vaginal discomfort after routine treatment of acute VVC and improve vaginal pH.	(De Seta et al., 2014)
Russo et al.	2019	The combination of a *Lactobacillus* mixture with lactoferrin is a safe and effective auxiliary method to reduce VVC symptoms and recurrence.	(Russo et al., 2019)
Oerlemans et al.	2020	*Lactobacillus* can be used for VVC treatment in the future.	(Oerlemans et al., 2020)
De Gregorio et al.	2020	Biosurfactants from *Lactobacillus crispatus* BC1 can interfere with the adherence of *Candida in vivo* and *in vitro*, indicating its potential as a preventive measure against *Candida* mucosal damage during VVC.	(De Gregorio et al., 2020)

The above experiments focused on the possibility of *Lactobacillus* to prevent VVC. In view of the great potential of *Lactobacillus*, whether other probiotics can be used for the treatment and defense of VVC deserves further research. In addition, it has been shown that certain biological components or subcomponents can inhibit the growth of *Candida*. de Freitas et al. used F2 and sub-fraction F2.4 tannins from Stryphnodendron adstringens stem bark to treat mice with vaginal infection with *Candida albicans* and analyzed vaginal histopathology and fungal load. The results showed that F2 and F2.4 have efficacy in controlling candidiasis in mouse models (de Freitas et al., 2018).

It should also be noted that there exist differences in the vaginal microbiome among women of different races, such as the body’s own immune system, the different amounts and composition of vaginal secretions. Therefore, it needs to be further studied whether treatment and defense of *Lactobacillus* and other microbiota against VVC are also related to the host’s genetic factors, behavior, and cultural differences (Denning et al., 2018).

## Conclusion

**Figure 2:**
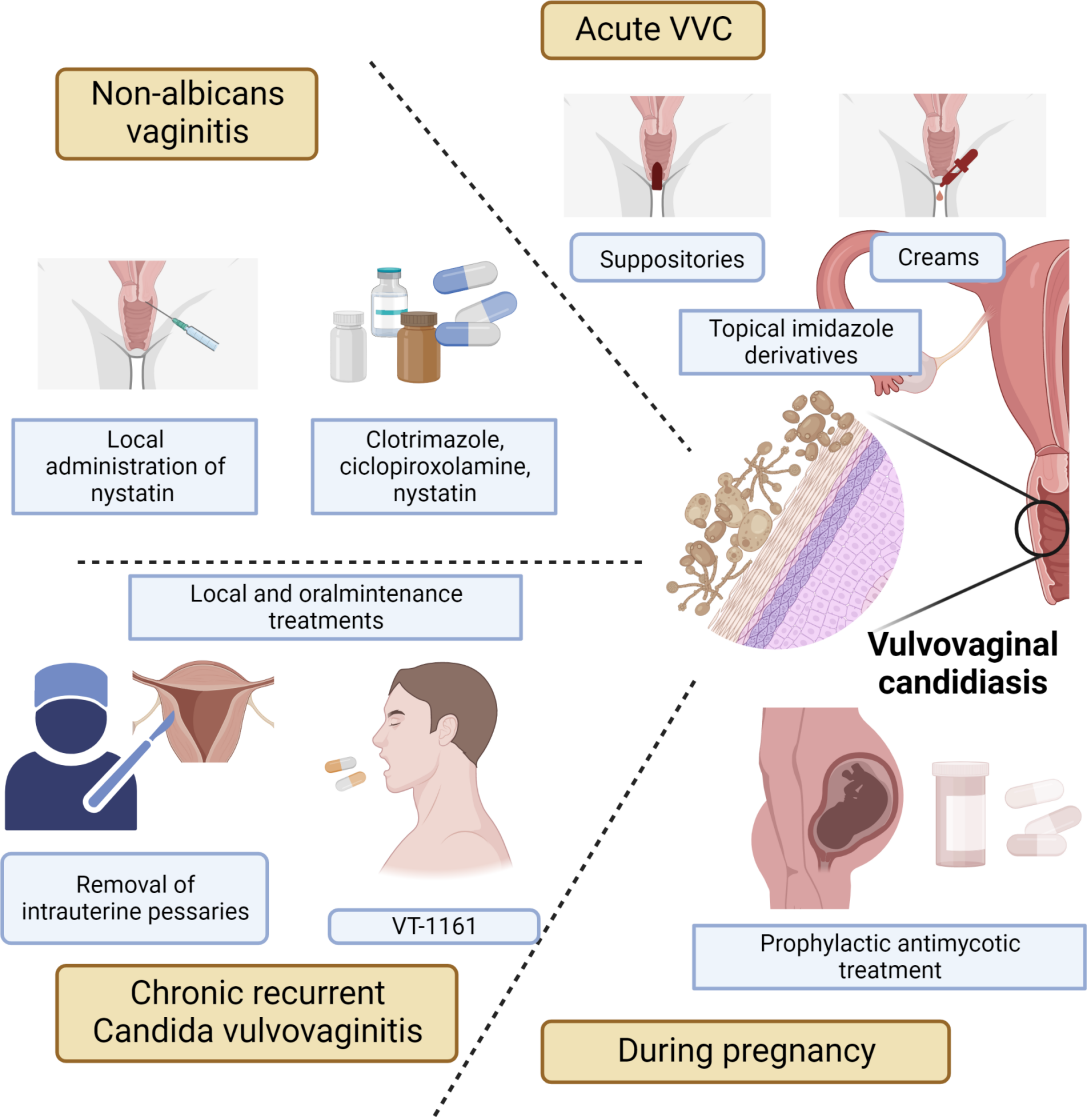
Treatments for acute VVC or recurrent VVC, including prescription oral formulations, over-the-counter topical preparations, and vaginal suppositories.

VVC is a complex disease, and its symptoms are affected by host physiology, fungal biology and immune response. Currently, there are many treatments for VVC, mainly including prescription oral dosage forms, over-the-counter topical preparations, and vaginal suppositories (**Figure 2**).

Given the projected global prevalence and economic burden of VVC in the next decade, high-income countries need better solutions and improved quality of care for affected women. With the increasing awareness of human microorganisms, probiotic treatment has been a hot topic in recent years. In many *in vivo* and *in vitro* experiments, *Lactobacillus* have been shown to have a certain effect on the prevention and treatment of VVC, but clinical data are still scarce and need to be further explored. It is believed that with the advancement of technology, the composition of vaginal microbiome and the preventive and therapeutic effects of vaginal microbiome on VVC can be further elucidated.

## Author contributions

ZS, XG and BQ had the idea for the article. ZX and CJ performed the literature search and data analysis. JW and YL drafted and critically revised the work. All authors contributed to the article and approved the submitted version.
